# Classroom Concordancing and Second Language Motivational Self-System: A Data-Driven Learning Approach

**DOI:** 10.3389/fpsyg.2022.841584

**Published:** 2022-04-15

**Authors:** Javad Zare, Sedigheh Karimpour

**Affiliations:** ^1^Department of English Language and Literature, Kosar University of Bojnord, Bojnord, Iran; ^2^Department of English Language, Mazandaran University of Medical Sciences, Sari, Iran

**Keywords:** data-driven learning (DDL), concordancing, motivation, L2 motivational self-system, EFL (English as a foreign language)

## Abstract

Research shows that exploring language corpora through data-driven learning (DDL) plays a significant role in language learning. Nevertheless, it is not clear if using concordancing as an application of DDL affects the learners’ second language motivation. To address this gap, the current study adopted a triangulation design, validating quantitative data model, and a quasi-experimental (comparison group, pretest-posttest) design. Ninety English-major university students with an intermediate level of English language proficiency, divided into control and experimental groups, took part in the study. Drawing on a second language motivational self-system questionnaire, the findings of the study did not result in any statistically significant differences between the students in their second language motivational self-system. Altogether, the students found learning English through a DDL approach with concordancing less motivating than receiving explicit instruction. The study has implications for language teaching and learning.

## Introduction

Over the past decades, researchers of the field have seen a rapid increase in empirical studies regarding the application and use of data-driven learning (DDL) and its’ role in second and foreign language learning (e.g., Gilquin and Granger, 2010; Boulton, 2017; Chen and Flowerdew, 2018). DDL provides a large number of instances of real context for the learners and creates a learning environment which attracts learners’ attention and thus enhances their learning (Geluso, 2013). Exploring language corpora through a DDL approach plays a significant role in language learning. It has been regarded as one of the most effective tools in second language learning development (e.g., O’Keeffe et al., 2007; Boulton, 2015). As Hunston (2002) stressed, “it is no exaggeration to say that corpora, and the study of corpora, have revolutionized the study of language, and of the application of language…” (p. 1). Through providing systematic access to naturally occurring language and corpus-linguistics methods, corpora and concordancing assist exploratory learning and hence, leads to autonomous learning and teaching (Kheirzade and Marandi, 2014).

The last two decades have seen a surge of interest in exploring the role of data-driven learning (DDL) in language teaching and learning (e.g., Yoon and Hirvela, 2004; Koosha and Jafarpour, 2005; Geluso, 2013; Yoon and Jo, 2014; Yunus and Awab, 2014; Zare et al., 2021). For example, the study by Koosha and Jafarpour (2005) on the use of DDL for teaching collocations of prepositions found DDL effective in enhancing students’ sentence production. Findings show the advantages of DDL through concordancing in teaching and learning academic skills and sub-skills and suggesting their efficiency and application in second language teaching and learning (Zare et al., 2021).

Despite the advantages of DDL and concordancing for foreign/second language learning, an extensive literature review indicates that there is no research on the impact of DDL and concordancing on the students’ motivation and more particularly the students’ second language motivational self-system as a predictor of learning outcomes. Thus, in order to understand their connection, the present study attempts to investigate whether using concordancing, as an application of DDL, affects the students’ second language motivational self-system.

## Literature Review

The advent of corpus-based approaches to language teaching within the past decades has transformed language teaching and learning as well as the teacher’s and the learner’s position (Flowerdew, 2009; McEnery and Xiao, 2011; Leńko-Szymańska and Boulton, 2015). The incipient appearance of data-driven learning (DDL) coincided with a paradigm shift from teacher-centered approaches to learner-centered approaches in language teaching. DDL is defined as an inductive approach that utilizes the tools and techniques of corpus linguistics for instructional objectives (Gilquin and Granger, 2010; Flowerdew, 2015). These pedagogical purposes consist of exposure to authentic language that leads to augmented awareness of regular language patterns, helping learners to identify errors in their writing by consulting corpora, and portraying learners as discoverers who directly explore the corpus to make inferences. As an inquiry-based approach, DDL has redefined teachers’ and learners’ roles. From this perspective, learners are regarded as an autonomous and active researchers (Leech, 1997) who are responsible for their learning, while the teacher plays the role of a facilitator who supports the learners by teaching them how to use concordancers to query corpus in order to extract grammatical rules and infer the meanings of words in contexts (Boulton, 2017).

Over the past decades, there has been a substantial surge of interest in corpus-driven approaches and the application of the findings of corpus-based empirical research to language pedagogy (McEnery and Xiao, 2011). The underlying rationale behind the burgeoning inclination to such approaches might be direct exposure to authentic language *via* concordancers in a controlled way that boosts noticing, consciousness-raising, autonomy, and agency (Boulton, 2017). Corpora are managed by concordancers. A concordancer is a tool, used to generate lists of linguistic data, known as concordance lines, for exploring how language works in different contexts. Concordancers can be used as a resource tool and act as a “linguistic informant” (Flowerdew, 1996, p. 92) to inform both teachers and learners of the lexical and grammatical features of language.

The nexus between DDL and concordancing has been highlighted in the literature (Flowerdew, 1996; Boulton, 2017). Boulton (2015) notes that DDL concerns the use of specifically designed concordancers to probe language corpora. A concordancer helps learners to investigate and infer rules through exposure to a multitude of examples in a natural context. Within a DDL approach, the learner is provided with the opportunity to search for vocabulary in authentic contexts and infer grammatical patterns while engaged in a discovery journey. In this regard, concordancing plays a facilitative role, as it empowers the learners to make their own decisions and move toward self-directed learning (Chambers and Kelly, 2004) that requires the teacher’s scaffolding.

Driving from Vygotsky’s (1978) socio-cultural theory, the concept of scaffolding has gained prominence in the language learning process. According to the socio-cultural theory of learning, knowledge is socially constructed through collaborative dialogue and negotiation where the teacher mediates in the form of scaffolding. Within this framework, the teacher plays a mediational role and scaffolds the learner to move from interpersonal stage to intrapersonal one to achieve independence and autonomy. Within the socio-cultural theory, the learner moves from other-regulation to self-regulation through collaboration with more knowledgeable others (teacher or peer), thus leading to independence and autonomy. A key element in this process is the scaffolding prompts that help the learner to be involved in a gradual process of knowledge-construction under the guidance of the teacher. This explanation advocates the teacher’s guidance and support while the learner is involved in using technology in order to learn a foreign language.

Therefore, there is a nexus between the socio-cultural theory of learning and DDL in which the teacher is repositioned as a facilitator whose job is to scaffold the students and to teach them how to work with concordancers in order to search corpora, whereas the learner assumes an active role whose job is to generate knowledge and solve the problems. Within this perspective, the learner is seen as a “self-reflective motivated and intentional agent” in the “fluid and complex system of social relations, activities, experiences and multiple micro- and macro-contexts” (Boulton, 2017, p. 220). Hence, motivation plays a crucial role in helping the learner move forward within the socio-cultural theory.

The interrelationship between second language acquisition and second language motivation, as well as the crucial role of motivation in impacting the learners’ language functioning, is a frequent theme in second/foreign language research (e.g., Dörnyei, 2009; Taguchi et al., 2009; Papi, 2010; Moskovsky et al., 2016; Safdari, 2019). Research studies have resulted in different conceptualizations of second/foreign language learning motivation. As the most influential model of second language motivation, the second language motivational self-system approach sought to incorporate “affective and emotional factors with cognition” (Ryan and Dörnyei, 2013, p. 91). The L2 motivational self-system approach is based on the premise that “the way in which people imagine themselves in the future plays an important role in energizing them in the present” (Dörnyei, 2009, p. 9). As Dörnyei (2009) states, the L2 motivational self-system “represents a major reformation of previous motivational thinking by its explicit utilization of psychological theories of the self, yet its roots are firmly set in previous research in the L2 field” (p. 21).

Influenced by a number of theories such as the socio-educational model (Gardner, 2010), self-discrepancy theory (Higgins, 1987) and selves theory (Markus and Nurius, 1986), the L2 motivational self-system consists of three main components, including the ideal L2 self, the ought-to L2 self, and the L2 learning experience (Dörnyei, 2009). As Dörnyei (2005) notes, the ideal L2 self represents “an ideal future self-representation of the individual as a user of the L2” (p. 91). The “aspirations toward realizing these desirable future images as a proficient L2 user” both initiate and sustain the learner’s motivation to learn the language (p. 91). The ideal L2 self thus represents the learners’ hopes and wishes. The ought-to L2 self, on the other hand, refers to the learners’ perceived obligations and responsibilities to others. It represents the expectations, made by significant others. And finally, the L2 learning experience deals with the learners’ perceptions of their success and failure in the previous and immediate learning environment (Ryan and Dörnyei, 2013). This concerns aspects of the teacher, the peers and the curriculum (Al-Hoorie, 2018). The L2 motivational self-system has thus reformulated the concept of language learning motivation as “a form of self-development or self-realization” (Ryan and Dörnyei, 2013, p. 92).

Over the past years, several studies have investigated the students’ L2 motivational self-system in a variety of learning environments and contexts (e.g., Al-Shehri, 2009; Csizér and Kormos, 2009; Ryan, 2009; Taguchi et al., 2009; Papi, 2010; Safdari, 2019; Papi and Khajavi, 2021). Dastgheib (1996), for example, investigated the undergraduate students’ attitudes and motivation toward learning a second language. The major finding of the study showed a significant correlation between the students’ attitudes toward learning English and their desire to learn English (Dörnyei, 2009). Sadighi and Maghsudi (2000) examined the university students’ L2 motivational self-system and showed how integratively-oriented students over-perform and get significantly better results than instrumentally-oriented students in taking the TOEFL exam. Matin (2007) investigated the motivational characteristics of university students in Iran and found that most of the students are equally motivated, based on instrumental and integrative reasons. In a comparative study, conducted by Taguchi et al. (2009), focusing on “the ought to L2 self” attribute of Dörnyei’s (2005) model, the findings revealed that the overall effect of this attribute on the learners’ motivated behavior is less than “the ideal L2 self.” Csizér and Kormos (2009) also examined the L2 motivational self-system and found that there is a positive interrelationship between the ought to second language self and the encouragements provided by the students’ parents in increasing the students’ second language motivation. Papi (2010) investigated Dörnyei’s L2 motivational self-system in relation to L2 anxiety and observed that all the attributes of Dörnyei’s tripartite L2 motivational self-system, though in different extents, motivate the learners to attempt more in learning English (p. 476). Safdari (2019) examined the effectiveness of a vision-based motivational program and found it greatly effective in enhancing the (L2) learners’ vision and motivation. The findings also showed that though the participants’ ideal L2 self toward L2 learning improves, their ought-to L2 self does not change (Safdari, 2019). More recently, Papi and Khajavi (2021) examined the motivational mechanisms underlying the second language achievement of 324 university students, using L2 motivational self-system. The findings of the study suggested that using a more promotion-oriented approach to language learning and teaching leads to more motivational, emotional, and behavioral patterns, which in turn contribute to the students’ language learning success.

Despite the importance of DDL and concordancing as an application of DDL in improving English language learning (Chambers and Kelly, 2004; Chambers, 2005, 2010; Boulton, 2009, Boulton, 2017; Gilquin and Granger, 2010; McEnery and Xiao, 2011; Flowerdew, 2015; Lee and Lin, 2019; Zare et al., 2021), no study has so far investigated the impact of using concordancing on the learners’ motivational behavior. Therefore, relying on Dörnyei’s tripartite model of the L2 motivational self-system, the present study aims to investigate whether using concordancing has any significant impacts on the students’ language learning motivation. In this respect, the present study attempts to answer the following research question:

Does concordancing, as an application of DDL significantly affect the students’ L2 motivational self-system and its attributes, including ideal L2 self, ought-to L2 self, and L2 learning experience?

## Materials and Methods

### Design

**FIGURE 1:**
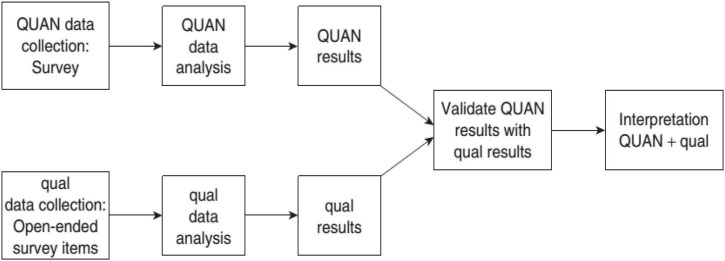
Triangulation design: validating quantitative data model [from Creswell and Plano Clark (2006)].

A triangulation design, validating quantitative data model, was adopted to better understand the impact of classroom concordancing as an application of DDL on the students’ L2 motivational self-system and enhance the credibility of the findings (Figure 1). Validating quantitative data model requires using both qualitative and quantitative types of data within one survey. The survey includes both quantitative and qualitative (open-ended) items. In this model, the qualitative data is used to validate and expand on the findings of quantitative data.

Within the validating quantitative data model, a quasi-experimental design, comparison group pretest-posttest, was used to explore the effect of classroom concordancing as an application of DDL on the students’ L2 motivational self-system. The design involved pretesting the students for L2 motivational self-system, assigning them into control and experimental groups, administering the treatment to the students in the experimental group and the control treatment to the students in the control group, and posttesting them on their L2 motivational self-system.

### Participants

A total of 312 English language learners volunteered to take part in the present study. From among them, 90 students with an intermediate level of English language proficiency were recruited for participation in the study. A stratified sampling procedure was followed to randomly select them based on their age, academic field of study, score on a Preliminary English Test (PET), score on an English academic lecture comprehension test, and score on an L2 motivational self-system questionnaire. They were all senior undergraduate students majoring in either English language teaching or English language and literature in Iran. The students were all female and their age ranged from 21 to 27 (*M* = 22.06, *SD* = 1.28). Moreover, the students were acquainted with concordancing, as they had all passed a course on technology-enhanced language learning before the study. The reason we selected such students was to minimize the novelty effect of using concordancing in the study.

### Instruments

The major instrument used in the present study to measure the students’ motivation and motivational attributes concerning English language learning was an L2 motivational self-system questionnaire. The L2 motivational self-system questionnaire was adapted from Dörnyei et al. (2006) and Taguchi et al. (2009). It comprised of three major sections in Persian. The first section consisted of 37 statement-type items measuring the students’ attitude and motivation toward English language learning with six-point Likert scales (strongly disagree, disagree, slightly disagree, slightly agree, agree, strongly agree). The second section consisted of 17 question-type items measuring their attitude and motivation concerning English language learning with six-point Likert scales (not at all, not so much, so-so, a little, quite a lot, very much). The third section consisted of one open-ended item asking the students to explain in great details how the intervention affected their English language learning motivation. Overall, the questionnaire measured the students’ L2 motivational self-system (α = 0.89) and its attributes, including ideal L2 self (α = 0.82), ought-to L2 self (α = 0.79), and L2 learning experience (α = 0.80).

A PET, piloted for the study with a reliability coefficient of 0.87, was used in the study. The PET was used to ensure that the participants have an intermediate level of English language proficiency. Additionally, an English academic lecture comprehension test, piloted for the study with a reliability coefficient of 0.80, was used to measure the students’ level of English academic lecture comprehension and ensure that the students have the same level of English academic lecture comprehension. The test had been developed by Zare (2020). It took 60 min to finish, comprising of 10 four-choice questions each asking about the important points of a short English academic lecture. A concordance, AntConc (3.5.8 for Windows) which is a freeware corpus analysis tool, was also utilized in the present investigation. AntConc was used as a concordancer to provide the students with access to the corpus.

### Procedures

The study consisted of three important phases: pretesting, administering the intervention, and posttesting. The first phase required using an L2 motivational self-system questionnaire, a PET, and an English academic lecture comprehension test to pretest the learners. The purpose of this part of the study was to ensure that the learners were all similar in terms of L2 motivational self-system, general English language proficiency, and English academic lecture comprehension. Pretesting the learners on their general English proficiency and English academic lectures comprehension was to exclude high and low achievers and make sure that having a high or low command of English doesn’t influence their L2 motivational self-system. Before administration, the students were told that their responses would only be used for research purposes. Upon the completion of pretest and assigning the learners to control and experimental groups, the second phase which was administration of the intervention was initiated. As the purpose of the study was to see if concordancing significantly affects the students’ L2 motivational self-system, two groups were formed, one experimental (45 participants) and one control group (45 participants). The students in the experimental group were exposed to the treatment and the ones in the control group were exposed to the control treatment. The treatment was a 12-session program of concordancing. Here, the students were supposed to work with AntConc which gave them access to the concordance lines regarding importance markers in 160 English academic lectures of the British Academic Spoken English (BASE) corpus, generated in a previous study of Zare and Keivanloo-Shahrestanaki (2017). The students were informed that, as part of a DDL approach, they were supposed to work with the concordancer to realize how importance is marked in English academic lectures.

On the other hand, the control treatment was a 12-session program of the explicit instruction of importance marking. During this program, the results of a corpus-driven study of Zare and Keivanloo-Shahrestanaki (2017) were explicitly taught to the students. The program helped the learners detect importance markers and distinguish important points from unimportant information in English academic lectures (e.g., *the point I am trying to make is*, *what I want you to have in mind is that*, *my point is*, and etc.). The medium of instruction for both the treatment and control treatment was Persian, though examples, exercises, and concordance lines were all in English. Upon the completion of the treatment and control treatment within 6 weeks, the L2 motivational self-system questionnaire was administered to the students in both groups to see if the intervention had any influence on the students’ L2 motivational self-system and its attributes.

### Statistical Analysis

Four separate Mann-Whitney *U*-tests were run, using the Statistical Package for Social Sciences (SPSS) software, one for L2 motivational self-system and the other attributes of the L2 motivational self-system. In these tests, classroom concordancing or explicit instruction was considered as the independent variable and the students’ L2 motivational self-system and its attributes, including ideal L2 self, ought-to L2 self, and L2 learning experience were the dependent variables.

## Results

Four separate Mann-Whitney *U*-tests were run to investigate if concordancing, as an application of DDL significantly affects the students’ L2 motivational self-system and its attributes. Table 1 presents the mean ranks for the students’ L2 motivational self-system and its attributes.

**TABLE 1:** The mean ranks for L2 motivational self-system and its attributes.

	Group	N	Mean rank	Sum of ranks
Ideal L2 self	Control	45	45.52	2048.50
	Experimental	45	45.48	2046.50
	Total	90		
Ought-to L2 self	Control	45	46.18	2078.00
	Experimental	45	44.82	2017.00
	Total	90		
L2 learning experience	Control	45	45.67	2055.00
	Experimental	45	45.33	2040.00
	Total	90		
L2 motivational self-system	Control	45	46.48	2091.50
	Experimental	45	44.52	2003.50
	Total	90		

As Table 1 shows, the mean ranks for the students’ L2 motivational self-system were different for the students in the control (*M* = 46.48, *N* = 45) and experimental (*M* = 44.52, *N* = 45) groups, with that of the students in the control group slightly higher. Also, the mean ranks for ideal L2 self, ought-to L2 self, and L2 learning experience were different for the students in the control (*M* = 45.52, 46.18, 45.67, *N* = 45) and experimental groups (*M* = 45.48, 44.82, 45.33, *N* = 45), with those of the students in the control group a little higher. In other words, in terms of L2 motivational self-system and its attributes, including ideal L2 self, ought-to L2 self, and L2 learning experience, the students in the control group were slightly more motivated to learn English than the students in the experimental group, following the intervention. As the differences were slight, the results of Mann Whitney *U* tests were considered. Table 2 presents the results of the four Mann-Whitney *U*-tests.

**TABLE 2:** The results of the Mann-Whitney *U*-tests for L2 motivational self-system and its attributes.

	Ideal L2 self	Ought-to L2 self	L2 learning experience	L2 motivational self-system
Mann-Whitney *U*	1011.500	982.000	1005.000	968.500
Wilcoxon W	2046.500	2017.000	2040.000	2003.500
Z	−0.008	−0.246	−0.061	−0.355
Asymp. Sig. (2-tailed)	0.994	0.806	0.952	0.722

As Table 2 shows, the “Sig. (2-tailed)” value of the students’ L2 motivational self-system and its attributes, including ideal L2 self, ought-to L2 self, and L2 learning experience were “0.722,” “0.994,” “0.806,” and “0.952,” respectively. Therefore, the female students’ L2 motivational self-system, and its attributes did not differ statistically significantly for the students in the control and experimental groups, following the intervention. In other words, using concordancing did not lead to any statistically significant influence on the female students’ L2 motivational self-system.

## Discussion and Conclusion

The present study sought to explore if concordancing, as an application of DDL significantly affects the female learners’ L2 motivation. To this end, four separate Mann-Whitney *U*-tests were run, one for L2 motivational self-system and one for each of the other attributes of the L2 motivational self-system. The findings of the study showed that in terms of L2 motivational self-system and its attributes, including ideal L2 self, ought-to L2 self, and L2 learning experience, the students in the control group were slightly more motivated to learn English than the students in the experimental group, following the intervention. In other words, the results of the study confirmed that using concordancing as an application of DDL did not statistically lead to any significant impact on the female students’ L2 motivational self-system. This could be related to the following points.

First, concordancing as an application of DDL seems to be a highly demanding and challenging experience which might have caused the students not to favor the approach very much. This demanding challenge led the students to reflect lower motivational, emotional, and behavioral patterns, which in turn might contribute to the students’ lower language learning success. This supports Vannestål and Lindquist (2007) and Boulton’s (2010) view that working with corpora, concordancers and DDL approach at the same time would be demanding for learners. Moreover, previous studies (e.g., Leńko-Szymańska and Boulton, 2015) confirmed that using corpus-based activities in a more deductive approach reduces the cognitive demands of DDL and it is due to the advanced technical skills concordancing requires for both teachers and learners that most language teachers are reluctant to use concordancing among their classroom activities (Leńko-Szymańska and Boulton, 2015). Thus it seems that most of language teachers and learners are not quite familiar with the application of DDL and concordancing.

Second, the absence of explicit instruction and the students’ inclination toward the teacher as an expert and a provider of knowledge might have decreased the students’ motivation slightly. As confirmed by previous studies (e.g., Grafton et al., 2014; Mobini et al., 2014), the teachers’ explicit instruction reduces anxiety symptoms among the students, and might thus increase the students’ motivation. This is in keeping with the findings of Zare et al. (2021) that using DDL through concordancing not only did not lead to increase in the students’ enjoyment of foreign language learning but also resulted in increase in their anxiety. Hence, the students’ less motivation might also be explained by their less foreign language enjoyment following the intervention. Moreover, the students’ lack of experience in concordancing and being novice to DDL could have potentially decreased their motivation. Concordancing demands a high level of computer literacy and autonomy which most undergraduate students lack.

The students in the control group in their responses to the open-ended item pointed to some factors that increased their learning motivation, including the good behavior of the teacher and the friendly atmosphere provided, the teacher’s expertise and skill in teaching, the teacher’s detailed and comprehensive explicit teaching, and feedbacks when it was demanded, and also the interesting and appropriate materials, used by the teacher.

The first factor which was reported by some students was the good behavior of the teacher and the friendly atmosphere the teacher provided in the classroom. As one of the students remarked:

*It seems that our teacher truly understands us and he is a very hardworking and generous teacher. He understands every aspect of our learning process and this increased my motivation.*

The second factor which was mentioned by the students was the teacher’s expertise and skill in teaching specific points and providing complete and comprehensive explanations for the new and important points. As one of the students stated:

*The teacher provided detailed explanation for every details of the lesson and I really enjoyed his teaching style and the way he provided comprehensive explanations for the new points increased my motivation.*

The third factor was appropriate teacher feedbacks. Some of the students reported that the feedbacks they received from their teacher at the appropriate time during the learning process increased their motivation. One of the students remarked:

*The feedbacks I received from my teacher at the appropriate time helped me to increase my motivation and helped me to answer many questions that I could not answer them without my teachers’ feedback.*

The fourth factor, mentioned by the students, to have increased their motivation was the use of novel, interesting and appropriate materials by the teacher. The students remarked that the materials were new and precise and this increased their motivation to some degree. As one of the students reported:

*I really enjoyed the new materials which my teacher used. This increased my motivation a lot and I really felt the need to learn more and to try more!*

Another student also remarked:

*The materials which the teacher used were brief and to the point and I really enjoyed them. They were totally understandable and I was motivated to know more about the details.*

On the other hand, the students in the experimental group in their responses to the open-ended item pointed to some factors that decreased their learning motivation, including lack of explicit teaching on the side of teacher, lack of teachers’ emotional support, lack of group activity and interaction between the students and finally, the non-conformity of the students’ individual learning style to the new style of teaching, DDL through concordancing, used by the teacher.

First and foremost, most of the students in the experimental group mentioned lack of explicit teaching as one of the major points which decreased their motivation. This is due to the students’ reliance on explicit instruction by the teacher and the fact that a DDL approach through concordancing was followed in the experimental group. As Grafton et al. (2014) note, the teachers’ explicit instruction has the potential to reduce the cognitive demands of DDL. In this regard, one of the students remarked:

*It is the teachers’ explicit instruction that causes learners to be interested in teaching materials and to be motivated. The teacher’s lack of explicit teaching decreased my motivation.*

The second point was the lack of the teachers’ emotional support. Boulton (2015) argued that the learners should take responsibility for their own learning instead of being reliant on the teacher as intermediary. However, it seems that the learners viewed their teachers’ as emotional supporter too. As one of the students noted:

*Teacher’s emotional support and explicit teaching could increase my motivation. But, there was no emotional support and teaching.*

Third, some of the students reported lack of interaction between the students as the reason for reduction in their motivation. This is in keeping with the fact that in concordancing, learning occurs through mediation by metatalk and is the consequence of interacting with corpora and concordances (Flowerdew, 2015). The students’ decrease in their motivation might have been due to the fact that they were not suited to this form of mediation. As one of the students pointed out:

*I think if I could interact with the other students through group work, this could increase our motivation.*

Fourth, some of the students noted that the new teaching approach was not appropriate for them and it did not match their individual leaning style, although some of them enjoyed using concordancers. As Flowerdew (2008) notes, corpus-based instruction advantages learners with various learning style preferences differently. In this respect, one of the students reported that:

*I don’t think that really helped me out with my motivation toward learning English but at some point it let me learn some tips. The problem was that it doesn’t match my learning style and I do not prefer it.*

Another student also pointed out:

*I really enjoyed learning English in in this course. It was interesting and I feel that it helped me know myself much more. I helped me be aware of my preferred learning style and made me be more curious.*

Overall, concordancing did not result in any statistically significant differences between the female students in their second language motivational self-system. That is, learning English through a DDL approach with concordancing did not motivate the female students. They even found learning English through a DDL approach with concordancing less motivating than receiving explicit instruction. Therefore, from the students’ perspective, engaging in DDL was not as effective and motivating as explicit instruction. Altogether, the results highlight the female students’ inclination toward teacher-centered classes, and their dependence on the teacher as the sole provider of information. Given the effectiveness of DDL though concordancing in improving the students’ comprehension of English academic lectures (e.g., Zare, 2020; Zare et al., 2021), it is important to raise the students’ awareness of the advantages of DDL and concordancing and encourage them to use it in learning certain aspects of the English language.

In order to reach a well-developed explanation of the impact of DDL and concordancing on the learners’ L2 motivational self-system, further replication research across different language learning contexts, language teaching methodologies, languages, aspects of the English language, and conceptualizations of the L2 learning motivation needs to be done. Hence, the results of the current analysis might be used for comparative purposes. Interested scholars are thus invited to do further research on these aspects. In so doing, using more process-oriented assessments of the students’ L2 learning motivation which is one of the limitations of the present study might also be insightful. The other limitation of the study, i.e., recruiting only female learners with an intermediate level of English language proficiency, also needs to be considered in future studies and larger samples of learners, including both male and female learners, with different proficiency levels should be studied.

## Data Availability Statement

The raw data supporting the conclusions of this article will be made available by the authors, without undue reservation.

## Ethics Statement

The studies involving human participants were reviewed and approved by the Kosar University of Bojnord Ethics Board. The patients/participants provided their written informed consent to participate in this study.

## Author Contributions

Both authors have contributed to the idea, data collection, analysis, and article preparation.

## Conflict of Interest

The authors declare that the research was conducted in the absence of any commercial or financial relationships that could be construed as a potential conflict of interest.

## Publisher’s Note

All claims expressed in this article are solely those of the authors and do not necessarily represent those of their affiliated organizations, or those of the publisher, the editors and the reviewers. Any product that may be evaluated in this article, or claim that may be made by its manufacturer, is not guaranteed or endorsed by the publisher.
